# Cancer, inflammation and the AT1 and AT2 receptors

**DOI:** 10.1186/1476-9255-1-3

**Published:** 2004-09-30

**Authors:** Gary Robert Smith, Sotiris Missailidis

**Affiliations:** 1 Research Department, Perses Biosystems Limited, University of Warwick Science Park, Coventry, CV4 7EZ, UK; 2 Chemistry Department, The Open University, Walton Hall, Milton Keynes MK7 6AA, UK

## Abstract

The critical role of inappropriate inflammation is becoming accepted in many diseases that affect man, including cardiovascular diseases, inflammatory and autoimmune disorders, neurodegenerative conditions, infection and cancer.

This review proposes that cancer up-regulates the angiotensin II type 1 (AT1) receptor through systemic oxidative stress and hypoxia mechanisms, thereby triggering chronic inflammatory processes to remodel surrounding tissue and subdue the immune system. Based on current literature and clinical studies on angiotensin receptor inhibitors, the paper concludes that blockade of the AT1 receptor in synergy with cancer vaccines and anti-inflammatory agents should offer a therapy to regress most, if not all, solid tumours.

With regard to cancer being a systemic disease, an examination of supporting evidence for a systemic role of AT1 in relationship to inflammation in disease and injury is presented as a logical progression. The evidence suggests that regulation of the mutually antagonistic angiotensin II receptors (AT1 and AT2) is an essential process in the management of inflammation and wound recovery, and that it is an imbalance in the expression of these receptors that leads to disease.

In consideration of cancer induced immune suppression, it is further postulated that the inflammation associated with bacterial and viral infections, is also an evolved means of immune suppression by these pathogens and that the damage caused, although incidental, leads to the symptoms of disease and, in some cases, death.

It is anticipated that manipulation of the angiotensin system with existing anti-hypertensive drugs could provide a new approach to the treatment of many of the diseases that afflict mankind.

## Review

### Tumour and Inflammation

Tumour has been linked with inflammation since 1863, when Rudolf Virchow discovered leucocytes in neoplastic tissues and made the first connection between inflammation and cancer [1]. Since then, chronic inflammation has been identified as a risk factor for cancer and even as a means to prognose/diagnose cancer at the onset of the disease. Examples of such association include the Human papiloma virus (HPV) and cancer [2], including cervical [3], cancers of the oesophagus [4] and larynx [5], Helicobacter pylori bacterial infection and gastric adenocarcinoma [6], the hepatitis B virus, cirrhosis and hepato-cellular carcinoma [7], Schistosoma haematobium and cancer of the bladder [8], asbestos induced inflammation and bronchogenic carcinoma or mesothelioma in humans [9].

Several reports implicate inflammation as a significant risk factor in cancer development: asbestos, cigarette smoke and inflammation of the bowel and pancreas are but a few well-known examples given [1,10]. These papers demonstrate that the inflammation environment is one that would support tumour development and is consistent with that observed in tumour sites. The relationship of cancer with inflammation is, however, not limited to the onset of the disease due to chronic inflammation. Schwartsburd [11] goes a step further and proposes that chronic inflammation occurs due to tumour environment stress and that this would generate a protective shield from the immune system. It has been recently demonstrated that the tumour microenvironment highly resembles an inflammation site, with significant advantages for the progression of tumour, including the use of cytokines, chemokines, leucocytes, lymphocytes and macrophages to contribute to both vassal dilation and neovascularisation for increased blood flow, the immunosuppression associated with the malignant disease, and tumour metastasis [1,11]. Furthermore, this inflammation-site tumour-generated microenvironment, apart from its significant role in cancer progression and protection from the immune system, has a considerable adverse effect to the success of the various current cancer treatments. It has recently been demonstrated that the inflammatory response in cancer can greatly affect the disposition and compromise the pharmacodynamics of chemotherapeutic agents [12].

It is evident that cancer is using natural inflammatory processes to spread and, unlikely as it seems at first, it is proposed that this is through the use of the angiotensin II type 1 (AT1) receptor.

### AT receptors and inflammation

Angiotensin II (Ang II) is a peptide hormone within the renin-angiotensin system (RAS), generated from the precursor protein angiotensinogen, by the actions of renin, angiotensin converting enzyme, chymases and various carboxy- and amino-peptidases [13]. Ang II is the main effector of the RAS system, which has been shown to play an important role in the regulation of vascular homeostasis, with various implications for both cardiovascular diseases and tumour angiogenesis. It exerts its various actions to the cardiovascular and renal systems via interactions with its two receptor molecules, angiotensin II type 1 receptor (AT1) and angiotensin II type 2 receptor (AT2) [13]. AT1 and AT2 receptors have been identified as seven transmembrane-spanning G protein-coupled receptors [13], comprising an extracellular, glycosylated region connected to the seven transmembrane α-helices linked by three intracellular and three extracellular loops. The carboxy-terminal domain of the protein is cytoplasmic and it is a regulatory site. AT1 is 359 amino acids, while AT2 is 363 amino acids being ~30% homologous to AT1 and are both N-linked glycosylated post-translationally. Various studies have looked at the pharmacological properties of the two receptors and the expression of those receptors on various cell lines. Their affinity for the angiotensin II peptide and their ability to perform their physiological functions has been characterised using radioligand binding analyses and Scatchard plots. The results have indicated that both receptors have high binding affinities for the AngII peptide. The AT1 receptor has demonstrated a Kd of 0.36 nM for the AngII peptide [14], whereas the AT2 receptor has demonstrated a Kd of 0.17 nM respectively, under similar studies [15].

AT1 receptors are expressed in various parts of the body and are associated with their respective functions, such as blood vessels, adrenal cortex, liver, kidney and brain, while AT2 receptors are highest in fetal mesenchymal tissue, adrenal medulla, uterus and ovarian follicles [13]. The opposing roles of the AT1 and AT2 receptors in maintaining blood pressure, water and electrolyte homeostasis are well established. It is, however, becoming recognised that the renin-angiotensin system is a key mediator of inflammation [16], with the AT receptors governing the transcription of pro-inflammatory mediators both in resident tissue and in infiltrating cells such as macrophages.

In addition to the mediators reviewed by Suzuki *et al *(2003) [16], a number of vital molecules in inflammatory processes are induced by the AT1 receptor. These include interleukin-1 beta (IL-1b) in activated monocytes [17], Tumour Necrosis Factor-alpha (TNF-α) [18], Plasminogen Activator Inhibitor Type 1 (PAI-1) [19] and adrenomedullin [20] all of which have been shown to have active participation in various aspects of cancer development. Activation of AT1 also causes the expression of TGF-β [21,22] and a review of literature indicates this may be a unique capability for this receptor. TGF-β is a multifunctional cytokine that is produced by numerous types of tumours and amongst its many functions is the ability to promote angiogenesis, tissue invasion, metastasis and immune suppression [23]. It has been postulated that the low response rates achieved in cancer patients undergoing immunotherapy is in part caused by tumour expression of TGF-β and this is supported by inhibition of the antigen-presenting functions and anti-tumour activity of dentritic cell vaccines [24].

On examination of the tumour environment, it is interesting to note that angiotensin II actually increases vasodilation, a phenomenon that researchers have attempted to utilise for drug delivery [25]. This would imply something unusual about the presentation of angiotensin receptors; however it is predominantly over expression of the vasoconstrictor AT1 that is reported in association with human cancers of the breast [26], pancreas [27], kidney [28], squamous cell carcinoma [29], keratoacanthoma [29], larynx [30], adrenal gland [30], and lung [31]. AT2 has been identified as expressed in preference to AT1 in only one case, in an earlier paper on colorectal cancer [32].

### Is it evolution that causes over expression of AT1?

In the 'Hallmarks of Cancer', the authoritative work by Douglas Hanahan and Robert A. Weinberg, the evolutionary acquired capabilities necessary for cancer cells to become life-threatening tumours are described. Furthermore, it is suggested that cancer researchers should look not just at the cancer cells, but also at the environment in which they interact, with cancers eliciting the aid of fibroblasts, endothelial cells and immune cells [33].

Sustained angiogenesis, tissue invasion and metastasis are the latter of six necessary steps in tumour progression, as described in the 'Hallmarks of Cancer' [33]. These envisaged evolutionary steps allow cancers to progress from growths of <2 mm to full tumors. A single evolutionary step, however, upregulation of AT1 would provide a considerable advantage to cancer cells that have learnt to evade the apoptosis and growth regulatory effects of TGF-β. Supporting this hypothesis is the observed genetic change from non-invasive cancer esophageal cell line T.Tn to invasive cancer cell line T.Tn-AT1. This genetic change concerns 9 genes, all of which are known to influence inflammation signalling (8 down and 1 up regulated) [34].

### Is it environment?

The alternative basis under which induction of AT1 in tumours may occur is by looking at the environment under which the cancer is developing. Stresses and cell damage on the growing tumour boundary could potentially be causing the expression of AT1. Evidence that appears to support this view can be found in a study of AT1 expression in breast cancers [35]. In this case, in situ carcinoma has over-expressed AT1 receptors in addition to expressing proteins for yet more AT1. In the invasive carcinoma, high proportions of AT1 receptors are found on the tumour boundary, but in this case protein generation for AT1 is very noticeably absent. How could this behaviour be explained? Perhaps the answer lies in oxidative stress and hypoxia.

The formation of oxidised LDL by monocytes and macrophages at the sites of tissue damage has been established in a recent report by Jawahar L. Mehta and Dayuan Li [36]. In this study, the ox-LDL LOX-1 receptor is noted to be induced by fluid shear stress (4 hrs), TNF-α (8 hrs) and self-induced by ox-LDL (12 hrs). Of particular interest is that activation of LOX-1 by ox-LDL induces the expression of the AT1 receptor [36]. This key role of ox-LDL regarding AT1 is demonstrated by HMG Co-A reductase inhibitor causing the down-regulation of the AT1 receptor with consequential reduction in inflammatory response [37]. Also of interest is that another marker of many diseases, homocysteine, enhances endothelial LOX-1 expression [38].

Hypoxia has been demonstrated to induce the expression of both AT1b (AT1a and AT1b are subsets of AT1) and AT2 receptors in the rat carotid body and pancreas [39,40]. The expression of AT1 and AT2 receptors has been studied during the development and regression of hypoxic pulmonary hypertension [41]. Hypoxia has been shown to strongly induce the expression of AT1b but not AT1a. The expression of AT2 is believed to protect the cell from apoptosis and this effect has been demonstrated in the brain when AT1 is antagonized [42]. Since HIF-1α governs many hypoxia driven transcriptions [43], its control of AT1b and AT2 expression can be hypothesized. AT1 activation has also been shown to increase the activity of HIF-1α [43], and is consistent with other cases of AT1 providing a positive feedback mechanism. Since hypoxia counts for the expression of AT1b, the speculation that the AT1a subtype is induced by oxidative stress is tempting, although a review of literature appears absent in this regard and further investigation is required to confirm this hypothesis.

**Figure 1:**
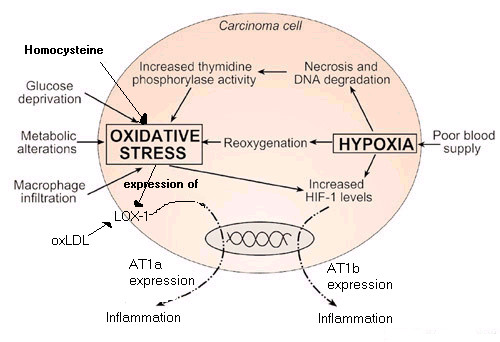
***AT1 expression in cancer. ***A cycle of oxidative stress (enhanced by homocysteine and ox-LDL) and hypoxia on the growing tumour boundary co-operatively promotes AT1 expression, leading to inflammation-associated angiogenesis, invasion, metastasis and immune suppression.

A review of hypoxia and oxidative stress in breast cancer cites the chaotic flow of blood in the tumor environment with resultant periods of hypoxia and reperfusion [44]. Reperfusion after myocardial infarction or cerebral ischemia is known to cause the generation of ROS. Hence, summarised in figure 1, the tumor environment thus offers both hypoxia and oxidative stress mechanisms for induction of AT1. It would however seem likely that genetic factors speed up the progression of the more aggressive forms of cancer.

### A combination therapy for cancer

The evidence relating to over-expression of AT1 with cancer progression is compelling. To this effect, AT1 blockade has been hypothesised as the mechanism to overcome cancer associated complications in organ graft recipients [45]. Additionally, a study undertaken in 1998 suggested that hypertensive patients taking ACE inhibitors were significantly less at risk of developing cancer than those taking other hypertensive treatments [46].

Tumour progression has been significantly slowed with AT1 receptor antagonists [47,48]. The results appeared to far exceed the expectations of simple inhibition of angiogenesis. Reduction of MCP-1 was noted [48], as was the expression of many pro-inflammatory cytokines. The activity of tumour-associated macrophages was also noticed to be severely impaired [48]. The importance in reducing the action of tumour-associated macrophages in extracellular matrix decomposition is not to be underestimated, since, in this action, they further progress remodelling by releasing stored TGF-β [49]. The similarity of action of tumour associated macrophages with those in the tissue healing and repair environment has been noted [49]. The tumour suppressant action of tranilast, an AT1 antagonist, [50] has been more widely explored [51-54]. In one study on the inhibition of uterine leiomyoma cells, Tranilast also induced p21 and p53 [55]. Similarly, the AT1 blocker losartan has been shown to antagonise platelets, which are thought to modulate cell plasticity and angiogenesis via the vascular endothelial growth factor (VEGF) [56]. It has been postulated that losartan and other AT1 blockers can act as novel anti-angiogenic, anti-invasive and anti-growth agents against neoplastic tissue [56]. Furthermore, it has been shown that angiotensin II induces the phosphorylations of mitogen-activated protein kinase (MAPK) and signal transducer and activator of transcription 3 (STAT3) in prostate cancer cells. In contrast, AT1 inhibitors have been shown to inhibit the proliferation of prostate cancer cells stimulated with EGF or angiotensin II, through the suppression of MAPK or STAT3 phosphorylation [57]. Angiotensin II also induces (VEGF), which plays a pivotal role in tumour angiogenesis and has been the target of various therapeutics, including antibodies and aptamers [58]. Although the role of angiotensin II in VEGF-mediated tumour development has not yet been elucidated, an ACE inhibitor significantly attenuated VEGF-mediated tumour development, accompanying the suppression of neovascularisation in the tumour and VEGF-induced endothelial cell migration [59]. Perindopril, another ACE inhibitor has also been shown to be a potent inhibitor of tumour development and angiogenesis through suppression of the VEGF and the endothelial cell tubule formation [60].

The powerful direct and indirect suppression effects of TNF-α [61], IL-1β [62] and TGF-β [63] on APC presenting cells, NK, T and B cell have been reviewed [64]. The expression of these mediators makes an effective immune response most unlikely.

Despite this, it has long been established that the body does have the capability to recognise cancer cells and develop antigens. Dentritic cell vaccines for instance have been developed and have demonstrated limited effect in treating established tumours. The effectiveness of one such approach was greatly enhanced leading to complete regression of tumours in 40% of cases when TGF-β was neutralised using TGF-β monoclonal antibodies in synergy with a dentritic cell vaccine [24].

Strong evidence suggests that tumour cells over-express AT1 receptors and compelling evidence has been presented on the implications of AT1 in cancer progression. Although still at a theoretical stage, this evidence leads to the formulation of the hypothesis that effective blockade of AT1 with a tight binding receptor antagonist, in combination with NSAIDs to further control the inflammation, and immunotherapy, such as cancer vaccines, would provide an effective treatment. Most, if not all, solid tumours utilise inflammation processes, which, through the over-expression and activation of AT1 and the subsequent expression of a number of inflammatory cytokines and chemokines, allow for tumour protection from the immune system through immunosuppression, as well as tumour progression and metastasis. Blocking these pathways through inhibition of AT1 using one of the commercially available AT1 inhibitors, whilst lifting the induced protective effect of immunosuppression and further reducing inflammation with the use of NSAIDs will both inhibit tumour progression and allow currently developed immunotherapies, such as cancer vaccines, to promote their therapeutic effect uninhibited. The role of AT1 post-metastasis, given the observation that AT1 protein expression ceases, as demonstrated in the breast cancer study, requires further investigation [35]. However, the premise for the necessity of immunosuppression by cancer is none the less fundamental and this is encouraging for the prospects of regression of cancers that have progressed to metastasis by combinational AT1 blockade/immune therapy.

### Learning from Cancer: wound management

Cancer is a systemic disease, one that can affect every part and organ in the body and, as presented in this review so far with regards to the role of AT1 in cancer, AT1 upregulation is of the utmost importance in the activation of inflammation. Systemically, therefore, what purpose does this upregulation of AT1 serve? The release of ACE and extended expression of AT1 and AT2 during the healing process following vascular injury helps to answer this question [65]. AngII is demonstrated to promote migration and proliferation of smooth muscle cells, as well as production of extracellular matrix through AT1 activation. In this work [65], the AT1 and AT2 receptors are recognized as having a substantial role in the tissue repair and healing processes of injured arteries. Although further literature in regard to the role of AT1 and AT2 in the healing process appears absent and additional studies are required, it appears rational that a systemic agent for the management of inflammation and healing would be one associated with the vascular system.

**Figure 2:**
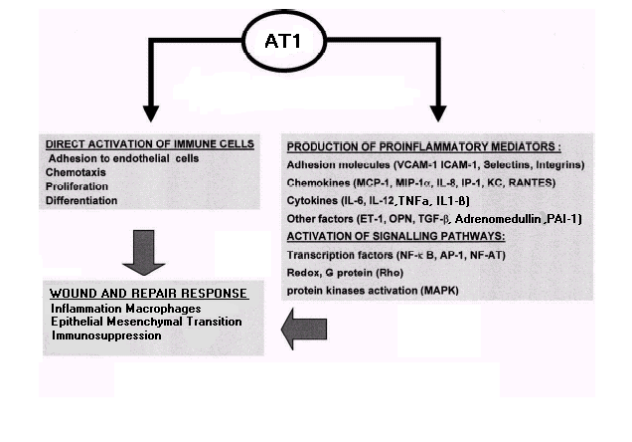
***AT1 signalling. ***Activation of AT1 has a powerful pro-inflammatory effect, promoting the expression of many pro-inflammatory mediators, such as cytokines, chemokines and adhesion molecules through the activation of signalling pathways. The influx, proliferation and behaviour of immune cells are steered away from an effective immune response to pathogens (thereby achieving immunosuppression) but instead towards activities consistent with a wound environment.

**Figure 3:**
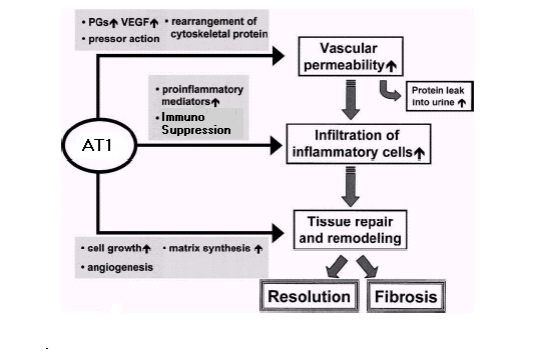
***local effects of AT1 activation. ***Activation of AT1 leads to growth factors causing increased vascular permeability, pro-inflammatory mediators that lead to utilisation of immune cells such as macrophages in their response to wound mode and other factors that promote cell growth, angiogenesis and matrix synthesis during fibrosis and resolution.

The activation of AT1 (shown in figure 2) has a powerful pro-inflammatory effect [16], promoting the expression of many pro-inflammatory mediators, such as cytokines, chemokines and adhesion molecules through the activation of signalling pathways. The influx, proliferation and behaviour of immune cells are steered away from an effective immune response to pathogens (thereby achieving immunosuppression) but instead towards activities consistent with a wound environment. Through the activation of these pathways [16], AT1 effectively elicits this response with local effects intended to initiate wound recovery through destruction of damaged cells, remodelling, the laying down of fibrous material and angiogenesis. AT1 acts in three ways, as indicated in figure 3. Firstly, via the up-regulation of growth factors that leads to increased vascular permeability. Secondly, through the increase of pro-inflammatory mediators that leads to utilisation of immune cells such as macrophages in their response to wound mode. Thirdly, through the generation of other factors which promote cell growth, angiogenesis and matrix synthesis. The observation that cancer resembles a wound that never heals is therefore substantiated.

### Confirming the systemic role of the AT1 receptor in inflammation and disease

With the role of AT1 in cancer established, when the literature of other diseases is reviewed, it is reasonable to anticipate that the role of this receptor is system-wide with regard to inflammation. Interest in the wider implications of the AT1 receptor within disease is gradually increasing and these studies further substantiate a systemic role for AT1 as a key inductor of inflammation and disease. In these studies, a wide variety of pro-disease mediators, such as TNF-α, NFκB, IL-6, TGF-β, surface adhesion molecules and PAI-I are shown to be induced by AT1 (Table 1).

**Table 1:** AT1 as a key inductor of inflammation and disease. A wide range of pro-inflammatory mediators, cytokines, chemokines and surface adhesion moleculesinvolved in a number of diseases are induced by AT1 and thus inhibited by its blockade.

**Disease**	**Mediators inhibited by AT1 blockade**	**Reference**
Cardiovascular disease	NFκB, 'markers of oxidation inflammation and fibrinolysis'	66
Cardiovascular disease	TGF-β	67
Cardiovascular disease	TNF-α, IL-6, ICAM-1, VCAM-1	18
Cardiovascular disease	PAI-1	19
Cardiovascular disease	Surface adhesion molecules	68,69
Cardiovascular disease	MCP in Hypercholesterolemia associated endothelial dysfunction	70
Kidney disease	None noted in this study.	71
Pancreatitis	(Key markers of the disease)	72
Liver fibrosis and cirrhosis	'TGF-β and pro-inflammatory cytokines'	21
Skin disease	None noted in this study.	73
Osteoporosis	'Markers of inflammation'	74
Alzheimer's, Huntington's and Parkinson's	(TGF-β [75], over expression of AT1 and AT2 noted in affected brain areas)	75–78

It is clear that a number of diseases, including heart and kidney disease, diseases associated with the liver and pancreas, as well as diseases of the skin, bone, the brain and most of the autoimmune and inflammatory disorders, are all affected by the AT1 blockade. It is worth noting at this stage that many of these diseases are often considered to be associated with ageing and with fibrosis. An investigation of the action of IGF-1 in the regulation of expression of AT2 leads to an explanation of this association.

### Role of IGF-1 in regulating AT receptors

The majority of studies on AT1 are related to cardiovascular disease, for which AT1 receptor antagonists were generated as treatment. Regarding AT2, although there has been increased research and interest in its role, this area appears little explored. That which has been learnt so far about the interplay and regulation of these receptors lends itself to a potentially useful model for the management of inflammation:

The expression of AT1 and AT2 receptors on fibroblasts present in cardiac fibrosis is investigated [79]. These types of fibroblast are noted for their expression of AT1 and AT2 receptors. The presence of IL-1b, TNF-α and lipopolysaccharides, through induction of NO and cGMP, all serve to down-regulate AT2 with no effect on AT1 leading to a quicker progression of fibrosis. Interestingly, the continuance in the presence of pro-inflammatory signals serves to delay expression of AT2. This is confirmed in a separate study of AT2 expression in proliferating cells. TGF-β1 and bFGF are shown as powerful inhibitors of AT2 expression, whilst IGF-1 is shown to induce the expression of AT2 [80].

IGF-1 is principally produced by the liver from GH (Growth Hormone) and circulates in the blood (decreasing with age) and is important in the regulation of immunity and inflammation [81]: IGF-1 is also capable of being produced by fibroblasts and macrophages on induction by pro-inflammatory cytokines, including TNF-α and IL-1b. In addition to the induction of AT2, IGF-1 can be seen as responsible for mediating the actions of many active cells in the immune/inflammation response [81]. Of note is that TNF-α and IL-1b also affect the circulating expression of IGF-1 by feedback on the release of GH from the anterior pituitary.

### The controlling role of AT receptors in inflammation and healing

Significant evidence has been shown that AT1 receptors are upregulated during disease and that AT2 receptor expression follows behind AT1 expression during injury and healing. Given the opposing roles of AT1 and AT2 it can thus be postulated that the interplay of these receptors plays a significant part in judging the current local status of appropriate versus inappropriate inflammation and in providing feedback to the rest of the body. Indeed it is anticipated that prolonged expression of AT1 combined with a lack of AT2 expression results in sustained chronic inflammation and fibrosis.

**Figure 4:**
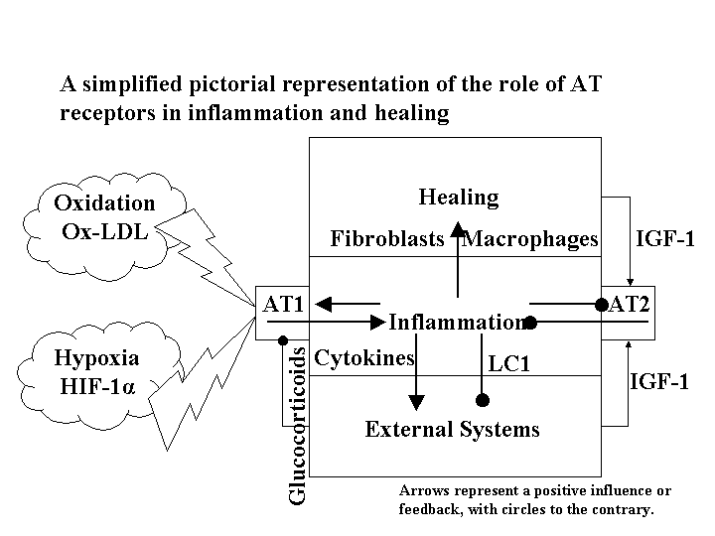
***Systems view of the AT receptor role. ***This hypothetical model shows the role of the mutually antagonistic AT receptors in managing levels of inflammation. Extended expression of AT1 in the absence of sufficient expression of AT2 may lead to a failure of inflammation resolution, sustained chronic inflammation and fibrosis. This model further serves to explain the pro-inflammatory role of hypoxia and oxidative stress and their risk factors in disease. Likewise the anti-inflammatory role of IGF-1 is supported and the risk factor of decreasing circulation of IGF-1 as a result of ageing. 'External Systems' represents non-local feedback i.e. the rest of the body including hypothalamus, pituitary, thyroid, adrenal glands, liver and pancreas.

Overall, the role of the AT receptors in managing and monitoring the healing process is complex, with many positive and negative feedback mechanisms both within the site of inflammation/healing and with the rest of the systems in the body. An attempt to summarise these systemic signalling inter-relationships is given in figure 4. Note the glucocorticoid inhibition of AT1 pro-inflammatory activities via NFκB. This model, although hypothetical, provides an explanation of the mechanisms whereby ox-LDL and homocysteine exert their pro-inflammatory effects. Further supporting this model is evidence that a lack of IGF-1 presence contributes to degenerative arthritis [81], septic shock [81], cardiovascular diseases [82] and inflammation of the bowel [83]. The introduction of IGF-1 is also proposed for protection against Huntington's [84], Alzheimer's [85] and Parkinson disorders [86]. Upregulation of IGF-1 has been noted in patients with chronic heart failure who undertake a programme of stretching exercise, thus providing benefits against cardiac cachexia [87].

## Conclusions

The invasiveness and immunosuppression of many cancers appears dependent on inflammation and the upregulation of AT1. Two mechanisms for upregulation of AT1 are discussed: 1) evolutionary changes to take advantage of this pro-inflammatory control mechanism, 2) AT1 expression induced by an alternating environment of hypoxia and oxidative stress. Immunosuppression as a common protection mechanism of solid tumours against immune responses has been verified from current literature and experimental procedures, as has the implication of cytokines and chemokines in tumour growth and metastasis. Given the involvement of AT1 in the immunosuppression and inflammatory processes, as well as in the expression of the pro-inflammatory cytokines and chemokines, it becomes evident that the AT1 receptor is essential for tumour protection and progression. A combination therapy consisting of AT1 receptor antagonists, NSAID for further control of the inflammation and immune therapy in the form of tumour vaccines should provide a novel and successful treatment for solid tumours.

In the renin-angiotensin system, the angiotensin II receptors AT1 and AT2 seem to have opposing functions. The actions of AT1 being principally pro-inflammatory whilst AT2 provides protection against hypoxia, draws inflammatory action to a close and promotes healing. The various direct and indirect mechanisms for feedback between the receptors, their induced products and the external hormonal system in the control of inflammation and healing are summarised in a highly simplified model which none the less can be used to explain how many key promoters and inhibitors of disease exert their effects.

From a review of the current disease literature, it has been demonstrated that the role of AT1 and AT2 in inflammation is not limited to cancer-associated inflammation, but is generally consistent and system wide. Potential therapy by manipulation of these receptors, although at an early stage, has been demonstrated for some of these diseases and it is proposed that this approach will provide an effective basis for the treatment of autoimmune, inflammatory and neurodegenerative disorders using existing drugs. AT1 receptor blockade should, in addition, provide a treatment to alleviate the damage caused by bacterial and viral infections, where their destructive action is through chronic inflammation. Given the importance of the immune suppressant effect of inflammation in cancer, it is anticipated that AT1 blockade should also serve to elicit a more effective immune response to other invaders that seek to corrupt the wound recovery process.

Manipulation of the AT1 and AT2 receptors has profound and exciting implications in the control of disease.

## List of abbreviations used

TGF-β Transforming Growth Factor Beta

AT1 Angiotensin II Type 1 receptor

AT2 Angiotensin II Type 2 receptor

IGF-1 Insulin-like Growth Factor 1

LOX-1 Lectin-like Oxidized Low-Density Lipoprotein Receptor 1

HIF-1a Hypoxia Induced Factor 1 Alpha

HMG CoA 3-Hydroxy-3-Methyl-Glutaryl Coenzyme A

bFGF basic Fibroblast Growth Factor

ROS Reactive Oxygen Species (most notably O_2_^-.^)

## Competing interests

Gary R Smith is a founding director of Perses Biosystems Ltd. The goals of the company are to drive laboratory and clinical research into the role of angiotensin receptors in disease management. Although we envisage these activities to be humanitarian (non-profit making) in nature, our long-term ambition is to identify additional drug targets and agents that could work in combination with ACE inhibitors and AT1 blockers to treat most diseases.

Sotiris Missailidis is a Lecturer at the Chemistry Department of The Open University, with research focus on cancer and had been the academic supervisor of Gary R Smith. There are no conflicting interests or financial implications related to the publication of this review article.

## Authors' contributions

Gary R Smith performed the literature review and proposed the hypothesis that cancer utilises the Angiotensin system to trigger chronic inflammation as a means of spreading and avoiding the immune system. In addition to providing significant editorial contributions and literature related comments, Sotiris Missailidis prompted Gary R Smith to undertake additional research with led to clarification of the role of hypoxia and oxidative stress in governing AT receptor expression. This understanding led Gary R Smith to propose the hypothesis that inflammation through the AT receptors is the cause of many of the diseases that affect mankind, including infectious diseases, which utilise inflammation to disrupt the immune system.
